# Modification of glucose import capacity in *Escherichia coli*: physiologic consequences and utility for improving DNA vaccine production

**DOI:** 10.1186/1475-2859-12-42

**Published:** 2013-05-02

**Authors:** Laura G Fuentes, Alvaro R Lara, Luz M Martínez, Octavio T Ramírez, Alfredo Martínez, Francisco Bolívar, Guillermo Gosset

**Affiliations:** 1 Departamento de Ingeniería Celular y Biocatálisis, Instituto de Biotecnología, Universidad Nacional Autónoma de México, Apdo. Postal 510-3, Cuernavaca, Morelos, 62210, Mexico; 2 Departamento de Procesos y Tecnología, Universidad Autónoma Metropolitana-Cuajimalpa. Artificios No. 40, Col. Miguel Hidalgo, Del. Álvaro Obregón, México, DF, CP 01120, Mexico; 3 Departamento de Medicina Molecular y Bioprocesos, Instituto de Biotecnología, Universidad Nacional Autónoma de México, Apdo. Postal 510-3, Cuernavaca, Morelos, 62210, Mexico; 4 Instituto de Biotecnología, Universidad Nacional Autónoma de México, Apdo. Postal 510-3, Cuernavaca, Morelos, 62210, Mexico

**Keywords:** *Escherichia coli*, Glucose transport, Phosphoenolpyruvate, Carbohydrate phosphotransferase system, DNA vaccine

## Abstract

**Background:**

The bacterium *Escherichia coli* can be grown employing various carbohydrates as sole carbon and energy source. Among them, glucose affords the highest growth rate. This sugar is nowadays widely employed as raw material in industrial fermentations. When *E. coli* grows in a medium containing non-limiting concentrations of glucose, a metabolic imbalance occurs whose main consequence is acetate secretion. The production of this toxic organic acid reduces strain productivity and viability. Solutions to this problem include reducing glucose concentration by substrate feeding strategies or the generation of mutant strains with impaired glucose import capacity. In this work, a collection of *E. coli* strains with inactive genes encoding proteins involved in glucose transport where generated to determine the effects of reduced glucose import capacity on growth rate, biomass yield, acetate and production of an experimental plasmid DNA vaccine (pHN).

**Results:**

A group of 15 isogenic derivatives of *E. coli* W3110 were generated with single and multiple deletions of genes encoding glucose, mannose, beta-glucoside, maltose and N-acetylglucosamine components of the phosphoenolpyruvate:sugar phosphotransferase system (PTS), as well as the galactose symporter and the Mgl galactose/glucose ABC transporter. These strains were characterized by growing them in mineral salts medium supplemented with 2.5 g/L glucose. Maximum specific rates of glucose consumption (*q*_*s*_) spanning from 1.33 to 0.32 g/g h were displayed by the group of mutants and W3110, which resulted in specific growth rates ranging from 0.65-0.18 h^-1^. Acetate accumulation was reduced or abolished in cultures with all mutant strains. W3110 and five selected mutant derivatives were transformed with pHN. A 3.2-fold increase in pHN yield on biomass was observed in cultures of a mutant strain with deletion of genes encoding the glucose and mannose PTS components, as well as Mgl.

**Conclusions:**

The group of *E. coli* mutants generated in this study displayed a reduction or elimination of overflow metabolism and a linear correlation between *q*_*s*_ and the maximum specific growth rate as well as the acetate production rate. By comparing DNA vaccine production parameters among some of these mutants, it was possible to identify a near-optimal glucose import rate value for this particular application. The strains employed in this study should be a useful resource for studying the effects of different predefined *q*_*s*_ values on production capacity for various biotechnological products.

## Background

**Figure 1:**
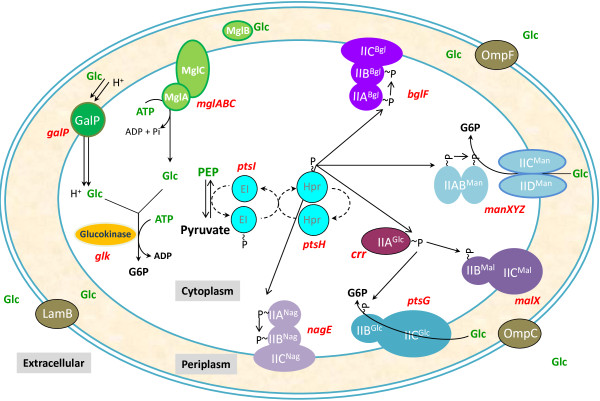
**Proteins involved in glucose transport and phosphorylation in *E. coli*. EI, enzyme I; HPr, phosphohistidine carrier protein; IICB**^**Glc**^**, integral membrane glucose permease; IIABC**^**Bgl**^**, components of the beta-glucoside PTS complex; IIAB**^**Man **^**and IICD**^**Man **^**components of the mannose PTS complex; IIBC**^**Mal**^**, components of the maltose PTS complex; IIABC**^**Nag**^**, components of the N-acetylglucosamine PTS complex; GalP, galactose:H**^**+ **^**symporter; MglA, MglB and MglC, components of the galactose/glucose high-affinity ABC transporter; LamB, OmpF and OmpC, outer membrane proteins.**

Biotechnological processes with *Escherichia coli* strains modified for protein, DNA or metabolite production frequently employ media containing glucose [1]. This carbohydrate is widely utilized as a raw material since it is relatively inexpensive and it is the preferred carbon and energy source for *E. coli* and other industrial microorganisms. In *E. coli*, glucose is internalized into the cytoplasm by the phosphoenolpyruvate:sugar phosphotransferase system (PTS). This is a complex protein system, widespread in bacteria and absent in Archaea and eukaryotic organisms [2]. PTS also participates in the transport and phosphorylation of other carbohydrates in addition to glucose. This system is composed of the soluble and non sugar-specific protein components enzyme I (EI) and the phosphohistidine carrier protein (HPr) that relay a phosphoryl group from phosphoenolpyruvate (PEP) to the sugar-specific enzymes IIA and IIB. PTS components IIC, and in some cases also IID, constitute integral membrane protein permeases that transports sugar molecules, which are phosphorylated by component IIB (Figure 1). In *E. coli*, 21 different enzyme II complexes have been identified that participate in import and phosphorylation of various carbohydrates [2]. Glucose is imported by the glucose enzyme II complex (II^Glc^) that includes the IIA^Glc^ enzyme and the integral membrane permease IICB^Glc^[3]. The mannose complex (II^Man^) is composed of the IIAB^Man^ enzyme and the integral membrane permease IICD^Man^. It has been reported that in addition to mannose, this PTS complex can also transport glucose [4]. An *E. coli* mutant strain with inactive glucose PTS complex still displays growth on glucose with a rate corresponding to about 80% of that observed in a wild type strain [5].

When *E. coli* grows under conditions of glucose limitation, proteins normally involved in galactose transport are induced and can also import glucose [6]. This physiological response has also been observed in *E. coli* strains with inactive PTS, even when growing in non-limiting glucose concentrations [7]. One of the genes induced under these conditions is *galP*, encoding the galactose:H^+^ symporter GalP [8]. The genes *mglABC*, encoding an ATP-binding protein, a galactose/glucose periplasmic binding protein and an integral membrane transporter protein, respectively, are also induced under glucose limitation. These proteins constitute the high-affinity ABC transporter Mgl system, involved in galactose/glucose transport [6]. The enzyme glucokinase, encoded by *glk*, catalyzes the ATP-dependent phosphorylation of the glucose molecules internalized by GalP or the Mgl system [9].

Under aerobic conditions when the glucose concentration in the medium is non-limiting, *E. coli* displays a high rate of substrate consumption and a corresponding high specific growth rate (*μ*). Under these conditions, *E. coli* secretes the organic acid acetate [10,11]. This is the result of a metabolic imbalance, also known as overflow metabolism, where the rate of acetyl-coenzyme A (AcCoA) synthesis surpasses the capacity of the tricarboxylic acid (TCA) cycle to completely consume this metabolite. Therefore, the amount of AcCoA not consumed by the TCA cycle is diverted into the phosphotransacetylase (Pta)-acetate kinase (Ack) pathway where acetate is synthesized [12]. Recent studies have proposed that acetate synthesis by the Pta-Ack pathway and its assimilation by AcCoA synthethase (Acs), constitute a futile cycle that maintains a physiological level of the regulatory metabolite acetyl-phosphate. A shift towards acetate accumulation occurs as a result of carbon catabolite repression of Acs expression under conditions of high glucose import rates [13,14]. Acetate production is a problem since this constitutes the loss of precursor molecule AcCoA. In addition, acetate is a toxic organic acid whose accumulation in culture media reduces productivity [15]. Solutions to this problem involve glucose feeding strategies to maintain a low concentration of this sugar in culture medium and also the generation of mutant strains with impaired acetate biosynthesis capacity [16,17]. In addition, mutant strains with reduced glucose import capacity have been generated with the purpose of decreasing acetate production. Inactivation of gene *ptsG*, encoding IICB^Glc^, resulted in a significant reduction of acetate secretion with a concomitant increase in production of a recombinant protein [5]. The complete inactivation of PTS by deletion of the *ptsHI-crr* operon encoding EI, HPr and IIA^Glc^ combined with the chromosomal overexpression of gene *galP*, encoding a non-PTS glucose importer, resulted in a strain displaying a PTS^–^ Glc^+^ phenotype, a lower rate of acetate production and a higher capacity for recombinant protein production [18].

One of the biotechnological products synthesized nowadays by recombinant *E. coli* is plasmid DNA (pDNA), which can be used as an alternative for gene therapy and immunization in various therapeutic applications [19]. Since plasmid titer is directly related to biomass formation, fermentation strategies for pDNA production are based on increasing cellular density. However, as stated above, acetate production can be an important drawback in this type of cultures. Some of the strategies employed to reduce acetate production in *E. coli* have been applied to pDNA production, with various degrees of success [19-21]. In the case of PTS modification, the use of a strain with a PTS^–^ Glc^+^ phenotype and reduced *q*_*s*_, resulted in increased production of pDNA [20].

The above examples illustrate how a *q*_*s*_ reduction in *E. coli* can have a positive effect on strain performance for the production of recombinant proteins and pDNA. However, previous works have focused on generating and studying either one or a few mutant strains with altered *q*_*s*_. It remains to be determined what would be the physiological and production performance effects of altering *q*_*s*_ over a wide range of values in a group of isogenic strains. With this purpose, a collection of *E. coli* mutants was generated with mutations in transporter proteins involved in glucose import. This group of 15 mutants and wild type W3110 span a *q*_*s*_ range from 1.33 to 0.32 g/g h, enabling a comparative study of the consequences of altering, within predetermined values, the glucose import capacity on key physiological parameters and the capacity to produce pDNA.

## Methods

### Strains and plasmids

The strains used in this work are described in Table 1. The reference strain *E*. *coli* W3110 is a derivative of *E. coli* K-12 [22]. Mutant derivatives of W3110 were constructed by P1 transduction, chromosomal gene inactivation employing PCR products or a combination of both methods [23]. Table 1 indicates the donor strains from the Keio collection that were employed as donors for P1 transduction experiments and the names of the oligonucleotides employed for PCR product gene inactivation [24]. To generate strains with more than one mutation, the gene encoding for kanamycin (Kan) resistance was excised employing plasmid pCP20 that expresses recombinase FLP [23]. Verification of the desired genotypes was made by PCR employing pairs of primers as follows: *ptsG*, ptsGF and ptsGR; *manX*, manXF and ManXR; *malX*, MalXF and MalXR; *bflF*, bglFF and bglFR; *nagE*, nagEF and nagER; *glK*, glk2F and glk2R; *galP*, galPF and galPR*; mglABC*, mglABF and mglABR; *ptsHIcrr*, PTSF and PTSR. Plasmid pcDNAHN176-construct (pHN) is an experimental vaccine against mumps. It contains the pUC origin of replication, an ampicillin resistance gene and a viral haemagglutinin-neuraminidase gene cloned under transcriptional control of the cytomegalovirus promoter [25].

**Table 1:** ***E. coli *strains and plasmid employed in this study**

**Name**	**Description**	Source or construction method*
**Strains**		
JW1087-2	F-, *Δ(araD-araB)567*, *ΔlacZ4787*(::rrnB-3), *λ*^*-*^, *ΔptsG763::kan*, *rph-1*,*Δ(rhaD-rhaB)568*, *hsdR514*	[23]
JW1806-1	F-, *Δ(araD-araB)567*, *ΔlacZ4787*(::rrnB-3), *λ*^*-*^, *ΔmanX741::kan*, *rph-1*,*Δ(rhaD-rhaB)568*, *hsdR514*	[23]
JW1613-1	F-, *Δ(araD-araB)567*, *ΔlacZ4787*(::rrnB-3), *λ*^*-*^, *ΔmalX769::kan*, *rph-1*,*Δ(rhaD-rhaB)568*, *hsdR514*	[23]
JW3700-1	F-, *Δ(araD-araB)567*, *ΔlacZ4787*(::rrnB-3), *λ*^*-*^, *rph-1*, *ΔbglF753::kan*,*Δ(rhaD-rhaB)568*, *hsdR514*	[23]
JW0665-1	F-, *Δ(araD-araB)567*, *ΔlacZ4787*(::rrnB-3), *ΔnagE728::kan*, *λ*^*-*^, *rph-1*,*Δ(rhaD-rhaB)568*, *hsdR514*	[23]
JW2910-1	F-, *Δ(araD-araB)567*, *ΔlacZ4787*(::rrnB-3), *λ*^*-*^, *ΔgalP789::kan*, *rph-1*,*Δ(rhaD-rhaB)568*, *hsdR514*	[23]
W3110	F^-^ λ^-^*rph-1 IN(rrnD-rrnE)*1	[23]
WG	W3110 ∆*ptsG*::FRT-Km-FRT	This study JW1087-2
WGX	WG ∆*malX*::FRT-Km-FRT	This study JW1613-1
WGB	WG ∆*bglF*::FRT-Km-FRT	This study JW3700-1
WGE	WG ∆*nagE*::FRT-Km-FRT	This study JW0665-1
WGM	WG ∆*manX*::FRT-Km-FRT	This study JW1806-1
WGMX	WGM ∆*malX*::FRT-Km-FRT	This study JW1613-1
WGMB	WGM ∆*bglF*::Km; FRT-Km-FRT	This study JW3700-1
WGME	WGM ∆*nagE*::FRT-Km-FRT	This study JW0665-1
WGP	WG ∆*galP*::FRT-Km-FRT	This study JW2910-1
WGC	WG ∆*mglABC*::FRT-Cm-FRT	This study mglABCDtF, mglABCDtR
WGMP	WGM ∆*galP*::FRT-Km-FRT	This study JW2910-1
WGMC	WGM, ∆*mglABC*::FRT-Cm-FRT	This study mglABCDtF, mglABCDtR
WHI	W3110 ∆*ptsHIcrr*::FRT-Cm-FRT	This study PTSDF PTSDR
WHIP	WHI ∆*galP*::FRT-Km-FRT	This study JW2910-1
WHIC	WHI ∆*mglABC*::FRT-Cm-FRT	This study mglABCDtF, mglABCDtR
W3110p	W3110/pHN	This study
WGp	WG/pHN	This study
WGMCp	WGMAB/pHN	This study
WGMp	WGM/pHN	This study
WGMEp	WGME/pHN	This study
WHICp	WHIAB/pHN	This study
**Plasmids**		
pHN	Derivative of pcDNA3.1 with cloned gene encoding for a 567 nucleotide region from the HN gene	[25]
pKD3	bla, FRT-Cm-FRT	[23]
pKD46	bla, γ β exo (red recombinase), temperature-conditional replicon	[23]
pCP20	bla, flp, temperature-conditional replicon	[23]
**Primers**		
ptsGF	CGC AGG TAA CCA CCG ATA AC	This study
ptsGR	GCA ACG CGC TAT ATT GCA GA	This study
manXF	ATC TGG CAC GTT GAC GTG TT	This study
manXR	TTG CCG TTA TCA GCA GCC TT	This study
malXF	AGC CAT GCA GAT GAC CTA CT	This study
malXR	AAC GGT CAG CGA CAT AAT CC	This study
bglFF	GGA TTG TTA CCG CAC TAA GC	This study
bglFR	AGG CAC CTT CCA CCT GAT TG	This study
nagEF	CGT TGG CGG ATT AGG CAT CT	This study
nagER	TGT TGG ATG CGA CGC TCA AG	This study
glk2F	CCG CCA GCA AGA CCG AGA AT	This study
glk2R	TCT ACC GCC GCT TCT TCC AG	This study
galPF	CGA TGC TGC CGG TCT GAA GT	This study
galPR	GTG TTG CGA CGC ACG GAT TG	This study
PTSF	CGA TGT GGC GGT AAC AAT CT	This study
PTSR	CCG CTT CAT AGC AGG TAT GT	This study
mglABF	GCT TCG GCG TTC AGT AAC AC	This study
mglABR	TAT GAC CGA ATG CGG ACC AC	This study
PTSDF	CTA GAC TTT AGT TCC ACA ACA CTA AAC CTA TAA GTT GGG GAA ATA CAA TGG TGT AGG CTG GAG CTG CTT C	This study
PTSDR	ATG GGC GCC ATT TTT CAC TGC GGC AAG AAT TAC TTC TTG ATG CGG ATA ACA TGG GAA TTA GCC ATG GTC C	This study
mglABCDtF	AGC ATT TAT CTC AAG CAC TAC CCT GCA TAA GAA AAA CCG GAG ATA CCA TGG TGT AGG CTG GAG CTG CTT C	This study
mglABCDtR	TTT ATG ACC GAA TGC GGA CCA CAT TCA CAT CAT TTC TTA CGC GCG TAT TTA TGG GAA TTA GCC ATG GTC C	This study

*The donor strain employed in P1 transduction or the names of the oligonucleotides employed for gene inactivation with PCR products are indicated.

### Growth media and cultivation conditions

The medium used during mutant strains construction and selection was Luria-Bertani (LB) with the corresponding antibiotic: chloramphenicol (Cm) 10 μg/mL, Km 10 μg/ml and carbenicilin (Cb) 100μg/mL. For shake flask cultures M9 mineral medium was used, containing 2.5 g/L glucose, 6 g/L Na_2_HPO_4_, 0.5 g/L NaCl, 3 g/L KH_2_PO_4_, 1 g/L NH_4_Cl, 0.5 g/L MgSO_4_•7H_2_O 0.01 g/L CaCl_2_ and 0.01 g/L thiamine hydrochloride. Cultures with strains for pHN production were performed in shake flasks with PD medium containing 2.5 g/L glucose, 17 g/L K2HPO4, 5.3 g/L KH2PO4, 2.5 g/L (NH4)2SO4, 1 g/L (NH4)Cl, 1 g/L sodium citrate, 1 g/L MgSO4 · 7H2O, 0.01 g/L thiamine hydrochloride and 2 mL of a stock solution of trace elements [20]. Glucose and salts solution were sterilized separately at 121°C during 20 minutes. All stock cultures were stored at −70°C in LB medium containing glycerol (40% v/v). The inoculum for shake flasks cultures consisted of 5 ml from LB overnight cultures that were sub-cultured in shake flasks containing 50 ml of M9 or PD medium with 2.5 g/L glucose starting with an OD_600nm_ = 0.1 and incubated at 37°C, 300 rpm in an orbital shaker until an OD of 2.0 was reached, then, a sample was used to inoculate shake flasks containing 50 mL of the same medium. Samples for analysis were taken periodically. The OD_600nm_ was determined and cells were centrifuged (13,000 g for 5 min) and the supernatant used for glucose and acetate determination by HPLC. In cultures for pHN production, an additional sample was taken to determine plasmid DNA concentration. The employed OD_600nm_ and cell dry mass (CDM) (g/L) correlation was CDM = 0.45 x OD_600nm_[26].

### Determination of glucose, acetic acid and plasmid concentration

The concentration of D-glucose and organic acids was determined employing an Aminex HPX-87H column (300 X 7.8 mm; 9 Am Bio-Rad, Hercules, CA). Separation was carried out isocratically with 5 mM H_2_SO_4_ at a flow rate of 0.5 mL/min and a temperature of 50°C. Under these conditions glucose was detected by refraction index and acetic acid by photodiode array at 210 nm. For these measurements a Waters HPLC system was used: 600E quaternary pump, 717 automatic injector, 2410 refraction index, and 996 photodiode array. pHN concentration was determined by extracting plasmid DNA with the Qiagen Spin Mini Prep kit (Hilden, Germany), following manufacturer instructions. The plasmid DNA concentration was determined at 260 nm using a Nanodrop UV spectrophotometer [27].

### Kinetic parameters calculation

For the characterization of the strains used in this work, maximum specific rates of growth (*μ*), glucose consumption (*q*_*s*_), acetate production (*q*_*ac*_), pHN plasmid production (*q*_*p*_), yield of biomass on glucose (Y_X/S_), yield of acetate on glucose (Y_ac/S_), yield of acetate on biomass (Y_ac/X_), yield of plasmid on glucose (Y_P/S_) and yield of plasmid on biomass (Y_P/X_) were determined. Yield values were calculated using linear regression of product concentration versus substrate or biomass concentration during the exponential growth phase. The linear least squares fit to the data displayed *R*^*2*^ values equal or higher than 0.97.

Values for *q*_*s*_, *q*_*ac*_, and *q*_*p*_ were calculated employing the following equations:

$$\begin{array}{l} q_{s}=m\mu /\left(\mathrm{Yx}s\right)\\ q_{\mathrm{ac}}=Y_{\mathrm{ac}/X}\cdot \;\mu \\ q_{p}=Y_{P/X}\cdot \;\mu \end{array}$$

These parameters were calculated during exponential growth phase. Flask cultures were performed in triplicate and the values reported represent the mean of all experiments.

## Results and discussion

### Generation and characterization of *E. coli* mutant strains lacking proteins involved in glucose import

The aim of this study was to generate and characterize a group of mutants derived from strain W3110, displaying a wide range of *q*_*s*_ values. The strategy followed to progressively decrease glucose import capacity consisted on inactivating genes encoding PTS components and non-PTS transport proteins. Under the growth conditions employed in this study (glucose 2.5 g/L), it can be expected that glucose is transported by the PTS, since the *K*_*M*_ of IICB^Glc^ for this sugar is 3 to 10 μM (0.6 to 18 mg/L) [3]. Therefore, mutants with inactive IICB^Glc^ and other PTS components were generated and characterized. It has been reported that IICD^Man^ can transport glucose in a strain lacking IICB^Glc^ activity [4]. However, it is not known which of the other remaining PTS complexes could be involved in glucose transport in the absence of IICB^Glc^ or in a double mutant lacking both IICB^Glc^ and IICD^Man^ components. In this study, we chose to inactivate selected components from the glucose and mannose PTS families, assuming they would show specificity towards glucose as substrate since both, IICB^Glc^ and IICD^Man^, can transport this sugar. Since the number of PTS complexes belonging to these two families is rather large, only a few members were arbitrarily selected for inactivation. Single, double and triple mutant derivatives of W3110 were constructed including genes encoding transport proteins from the glucose (*ptsG*), mannose, (*manX*), beta-glucoside (*bglF*), maltose (*malX*) and N-acetylglucosamine (*nagE*) PTS complexes. Inactivation of *ptsG* (strain WG) caused a 21%, 22% and 63% reduction in *q*_*s*_, *μ* and *q*_*ac*_, respectively (Table 2 and Additional file 1: Figure S1). This is expected since IICB^Glc^ is the main glucose importer under the studied conditions. However, these results also show that there is still significant remaining glucose import capacity in the mutant strain; therefore, other transport proteins are involved in this process. To generate mutant derivatives of strain WG with further reduced *q*_*s*_, genes encoding specific components of the above-mentioned PTS complexes were inactivated. In the *ptsG*^-^ background, inactivation of *malX* (strain WGX), *nagE* (strain WGE) or *manX* (strain WGM) caused a further reduction in *q*_*s*_ and *μ*. Furthermore, no acetate was detected in the supernatants of cultures with these three strains, indicating the elimination of overflow metabolism (Additional file 1: Figure S1). In contrast, inactivation of *ptsG* and *bglF* (strain WGB) did not decrease *q*_*s*_, when compared to strain WG. These results indicate that mannose, maltose and N-acetylglucosamine PTS complexes contribute to glucose import in the absence of IICB^Glc^. From strain WGM, mutant derivatives were generated to determine if beta-glucoside, maltose or N-acetylglucosamine PTS complexes participate in glucose transport in this genetic background. From these mutant strains, only WGME showed a reduction in *q*_*s*_ and *μ* of 58% and 57%, respectively, when compared to W3110.

**Table 2:** Kinetic and stoichiometric parameters of wild type and mutant strains lacking various glucose transporters

**Strains**	**Deleted gene(s)**	***μ *(h**^**-1**^**)**	***q***_***s ***_**(g/g h)**	***q***_***ac ***_**(g/g h)**	**Y**_**X/S**_
**W3110**	-	0.65 ± 0.02	1.33 ± 0.04	0.19 ± 0.02	0.49 ± 0.00
**WG**	*ptsG*	0.51 ± 0.01	1.05 ± 0.12	0.07 ± 0.05	0.53 ± 0.12
**WGX**	*ptsG, malX*	0.23 ± 0.03	0.51 ± 0.01	0	0.41 ± 0.01
**WGB**	*ptsG, bglF*	0.43 ± 0.04	1.06 ± 0.03	0.06 ± 0.02	0.42 ± 0.03
**WGE**	*ptsG, nagE*	0.41 ± 0.02	0.69 ± 0.06	0	0.60 ± 0.08
**WGM**	*ptsG, manX*	0.36 ± 0.03	0.65 ± 0.05	0	0.56 ± 0.08
**WGMX**	*ptsG, manX, malX*	0.32 ± 0.01	0.58 ± 0.06	0	0.55 ± 0.06
**WGMB**	*ptsG, manX, bglF*	0.29 ± 0.00	0.66 ± 0.07	0	0.40 ± 0.02
**WGME**	*ptsG, manX, nagE*	0.28 ± 0.02	0.56 ± 0.00	0	0.48 ± 0.03
**WGP**	*ptsG, galP*	0.49 ± 0.08	1.19 ± 0.00	0.06 ± 0.00	0.44 ± 0.01
**WGC**	*ptsG, mglABC*	0.48 ± 0.01	1.02 ± 0.00	0.08 ± 0.00	0.47 ± 0.05
**WGMP**	*ptsG, manX, galP*	0.29 ± 0.01	0.56 ± 0.01	0	0.52 ± 0.02
**WGMC**	*ptsG, manX, mglABC*	0.31 ± 0.01	0.68 ± 0.00	0	0.47 ± 0.01
**WHI**	*ptsHIcrr*	0.25 ± 0.01	0.49 ± 0.03	0	0.51 ± 0.02
**WHIP**	*ptsHIcrr, galP*	0.18 ± 0.02	0.32 ± 0.02	0	0.55 ± 0.03
**WHIC**	*ptsHIcrr, mglABC*	0.20 ± 0.01	0.32 ± 0.02	0	0.38 ± 0.05

Previous results indicated that strain WGME displays growth on glucose even after the inactivation of several transport proteins. Glucose transport in this strain could be dependent on both PTS and non-PTS transport proteins. To determine the extent of the contribution to glucose transport of the still-active PTS complexes in strain WGME, a mutant derivative of W3110 was generated by deletion of the *ptsHI*, -*crr* operon (strain WHI). Deletion of genes encoding EI and HPr disrupts the phosphotransfer chain, thus rendering all PTS complexes inactive. As expected, the complete elimination of PTS activity caused a severe reduction in *q*_*s*_ and *μ* of 64% and 62%, respectively, when compared to W3110. When compared to WGME, strain WHI showed 12% and 14% reduction in *μ* and *q*_*s*_, respectively. These results suggest that approximately 14% of the glucose import capacity in WGME is dependent on one or several of the still active PTS complexes in this strain, and the remaining capacity is dependent on non-PTS transporters. However, this conclusion should be taken with caution, since PTS is involved in regulatory processes that include genes encoding non-PTS transporters [28]. Therefore, it cannot be ruled out that altered regulatory responses on genes encoding non-PTS transporters could occur in strain WHI, when compared to WGME.

The transporter GalP and the Mgl protein system are known to participate in glucose import under conditions of glucose starvation. Even though the culture medium employed in this study contains a non-limiting glucose concentration, as a result of the inactivation of glucose importers, a low intracellular level of glucose-6-phosphate and other central metabolism intermediates could be expected in the mutant strains displaying low *q*_*s*_ values. To determine if GalP or Mgl are involved in glucose transport in some of these strains, derivatives lacking either one of these two importers were generated from WG and WGM. Strains WGP and WGC showed similar *q*_*s*_ and *μ* values as progenitor strain WG, indicating that GalP or Mgl do not contribute to glucose import in this genetic background. Whereas in the WGM background, strain WGMC did not show a change in glucose import capacity, while mutant WGMP displayed a decrease in *q*_*s*_ and *μ*. These results show that only after the *q*_*s*_ decreases to a value close to 0.65 g/g h, then a contribution of GalP to glucose import can be detected. To determine the involvement of non-PTS transporters on glucose import capacity in strain WHI, derivatives with inactive *galP* (WHIP) or *mglABC* (WHIC) were generated and characterized. Both mutants displayed a 35% reduction in *q*_*s*_ when compared to progenitor WHI. These results indicate that both GalP and the Mgl system contribute to glucose import in the strain lacking functional PTS.

**Figure 2:**
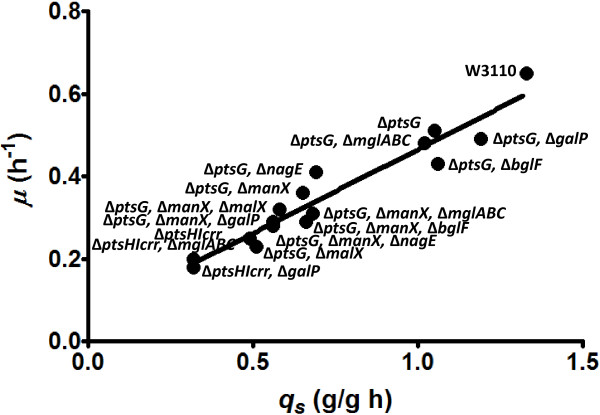
**Specific growth rate as a function of the specific glucose uptake rate for various *E. coli *strains generated in this study.**

When comparing the Y_X/S_ values among wild type and mutant strains, minimal differences were detected. The group of strains characterized in this study, including wild-type W3110, display a wide range of *q*_*s*_, *μ* and *q*_*ac*_ values. Under the conditions employed here, *μ* correlated linearly with *q*_*s*_ (*R*^2^ = 0.92) (Figure 2). This result is consistent with the small differences in Y_X/S_ among strains. As result of the distinct *q*_*s*_ values displayed by these strains, the amount of time taken to completely consume the 2.5 g/L of glucose in the culture medium varied from 6 to 28 h (Additional file 1: Figure S1). In addition to the physiological effects of such glucose consumption rates, these differences would be expected to have an impact on strain production capacity, mainly specific productivity, for various biotechnological products, as will be discussed below.

**Figure 3:**
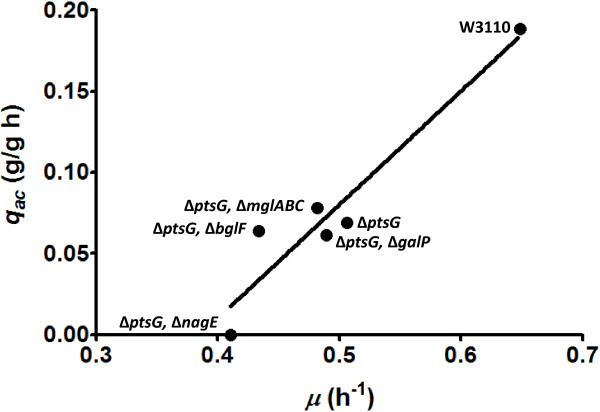
**Specific acetate production rate as a function of the specific growth rate for various *E. coli *strains generated in this study.**

When considering data from the five strains that accumulated acetate in the medium (W3110, WG, WGB, WGP and WGC) and WGE, the mutant strain with the highest *q*_*s*_ and *μ* values that did not produce acetate, it was found that *q*_*ac*_ correlated linearly with *μ* (*R*^2^ = 0.91) (Figure 3). It is not possible to ascertain if the observed correlation is maintained at *μ* values lower than 0.43 h^-1^ (strain WGB), nevertheless, linear regression analysis of this data was employed to calculate *μ* = 0.39 h^-1^ as the value corresponding to a *q*_*ac*_ = 0. This value is close to a *μ* of 0.375 h^-1^, that was determined from glucose-limited continuous cultures with *E. coli* K-12 TG1, as the lowest value where acetate was detected [29]. A study employing accelerostat cultivations with strain MG1655 identified a two-phase acetate accumulation pattern [13]. The slow rate acetate accumulation phase started at a *μ* of 0.27 h^-1^, whereas high rate acetate accumulation was detected at a *μ* of 0.46 h^-1^. Assuming a similar physiological response between strains MG1655 and W3110, it is possible that acetate accumulation in this study with mutant derivatives was detected until the high rate production phase was reached. Further detailed analyses, employing accelerostat or chemostat cultivations, should allow determination of the specific acetate accumulation pattern for each mutant strain.

The qualitative effects on *q*_*s*_ and *μ* of deleting specific PTS and non-PTS transporters in strain W3110 are in agreement with results reported with mutant derivatives of *E. coli* strains MG1655 and AF1000 having some of the same gene deletions performed in this study [30-32]. However, the magnitude of *q*_*s*_ reduction as function of specific gene inactivation differs from our results. This can be explained considering that different culture media was employed and the strains genetic backgrounds are not identical.

### Generation and characterization of strains with modified glucose import capacity for the production of a DNA vaccine

**Figure 4:**
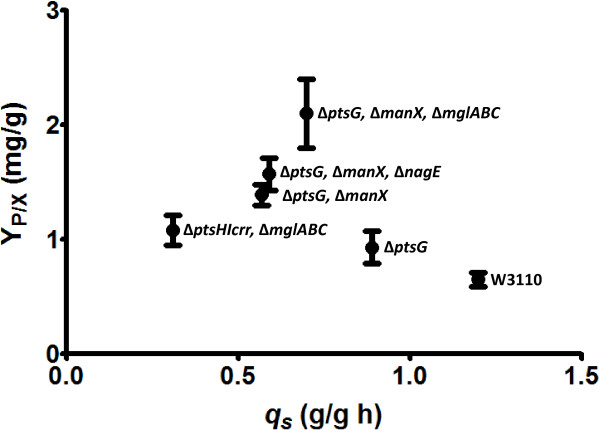
**Plasmid pHN yield from biomass as a function of the specific glucose uptake rate for various *E. coli *strains generated in this study.**

The characterization of the strains generated in this study enabled the identification of mutant derivatives of W3110 with distinct *q*_*s*_, and *q*_*ac*_ values. In several of these strains, acetate production was reduced or absent. Lower acetate production and accumulation is associated with an increased capacity for pDNA production in *E. coli* cultures [19,20]. To determine the effect of different *q*_*s*_, *q*_*ac*_ and *μ* values on pDNA production capacity, some of the strains generated in this study were transformed with plasmid pHN. Strains W3110, WG, WGM, WGME, WGMC, WHI and WHIC were transformed with plasmid pHN to generate recombinant strains W3110p, WGp, WGMp, WGMEp, WGMCp, WHIp and WHICp, respectively. These strains were grown in shake-flask cultures with PD medium supplemented with 2.5 g/L glucose. This culture medium has been employed previously for pDNA production studies; therefore, it was used here to facilitate strain performance comparisons. During strain characterization, it was determined that strain WHIp did not grow in PD medium; therefore, it was not considered for further analysis. Table 3 summarizes the kinetic and stoichiometric parameters of the six remaining strains. Among these strains, acetate was detected only in cultures with W3110 (0.4 g/L) (Additional file 2: Figure S2). The maximum pHN titer level varied among strains, with WGMCp displaying the highest value. The same trend was observed for plasmid yield from glucose (Y_P/S_) or from biomass (Y_P/X_). The highest plasmid specific productivity was observed for strain WGMCp and also for WGMp. Figure 4 shows the Y_P/X_ as a function of *q*_*s*_ for the pHN producing strains. Compared to strain W3110p, the Y_P/X_ for strains WGp and WGMCp were 1.4 and 3.2-fold higher, respectively. In the case of strain WGp, the observed increase can be explained by the lack of acetate production, when compared to W3110p. A similar result has been reported when comparing wild type and mutants defective in glucose transport in the case of recombinant protein production [18,31,33]. The final acetate titer in cultures with W3110p was not high enough to expect a toxic effect [15]. Therefore, the increase in pHN production in strain WGp can be attributed mainly to the elimination of overflow metabolism, resulting in a more efficient use of the carbon source. However, the higher Y_P/X_ observed for WGMCp, when compared to WGp, cannot be explained by lower acetate production, since this metabolite was not detected in culture supernatants of either strain. Alterations to glucose consumption, either by the inactivation of glucose importers or feeding strategies to limit this nutrient, have been known to alter central carbon metabolism fluxes [26,34]. It can be speculated that metabolic flux distribution in WGMCp favors the synthesis of DNA precursors, leading to higher pHN production. A more detailed comparative analysis of these strains, including fluxome and other “omics” approaches should allow a better understanding of the observed results.

**Table 3:** Kinetic and stoichiometric parameters of wild type and mutant strains transformed with plasmid pHN

**Parameter**	**W3110p**	**WGp**	**WGMCp**	**WGMp**	**WGMEp**	**WHICp**
		**(Δ *ptsG *)**	**(Δ *ptsG *, Δ *manX, * ΔmglABC)**	**(Δ *ptsG *, Δ *manX *)**	**(Δ *ptsG *, Δ *manX *, Δ *nagE *)**	**(Δ *ptsHIcrr *, Δ *mglABC *)**
Maximum biomass concentration (g/L)	1.14 ± 0.15	1.42 ± 0.02	0.82 ± 0.30	1.08 ± 0.05	1.12 ± 0.06	1.13 ± 0.16
Maximum plasmid pHN concentration (mg/L)	0.83 ± 0.18	1.06 ± 0.19	1.63 ± 0.45	1.09 ± 0.34	1.19 ± 0.41	1.47 ± 0.30
Maximum acetate concentration (g/L)	0.40 ± 0.01	0	0	0	0	0
*μ* (h^-1^)	0.63 ± 0.04	0.49 ± 0.00	0.33 ± 0.06	0.33 ± 0.02	0.37 ± 0.01	0.14 ± 0.02
Y_P/S_ (mg/g)	0.43 ± 0.15	0.54 ± 0.10	1.03 ± 0.01	0.89 ± 0.00	0.75 ± 0.05	0.84 ± 0.04
Y_P/X_ (mg/g)	0.65 ± 0.04	0.93 ± 0.14	2.10 ± 0.30	1.57 ± 0.14	1.39 ± 0.09	1.08 ± 0.13
*q*_*s*_ (g/g h)	1.20 ± 0.07	0.89 ± 0.07	0.70 ± 0.09	0.59 ± 0.03	0.57 ± 0.03	0.31 ± 0.07
*q*_*p*_ (g/g h)	0.50 ± 0.09	0.43 ± 0.02	0.53 ± 0.09	0.53 ± 0.05	0.51 ± 0.02	0.15 ± 0.01
Culture time (h)	5	7	9	8	9	24

A recent study has determined pHN plasmid productivity with strain DH5α, a high-performance plasmid production strain, under culture conditions similar to those employed here. This report revealed an Y_P/X_ of 6.56 ± 0.17 mg/g and a *q*_*p*_ of 2.59 ± 0.12 (g/g h) for pHN [27]. These values are approximately 3 and 5-fold higher for Y_P/X_ and *q*_*p*_, respectively, when compared to results with strain WGMCp. It should be pointed out, though, that strain DH5α bears several mutations that have the effect of increasing plasmid yield and productivity. It can be expected that these mutations would also increase plasmid production capacity in strain WGMCp, although the magnitude of such improvement cannot be estimated and would have to be determined experimentally.

Compared to WGMCp, strains with progressively lower *q*_*s*_, WGMp, WGMEp and WHICp displayed a lower Y_P/X_. It can be assumed that a low glucose consumption rate should cause a limitation of central carbon metabolism precursors and energy, having a negative impact on pHN production. A similar result has been reported when comparing two mutant strains for the production of a recombinant protein. A Δ*ptsG* Δ*manX* mutant produced less acetate than a Δ*ptsG* mutant, but unexpectedly, also less recombinant protein [33]. In this case, the authors point out that not only is low acetate production important for strain performance, but also an adequate cellular energy status.

The control of plasmid replication is a complex process influenced by the cell physiologic state. Growth rate is one of the parameters that have an impact on plasmid copy number. For plasmids having a pUC origin of replication, such as pHN, a higher copy number is observed when comparing slow to fast growing cells [35]. It has been generally accepted that plasmid copy number in inversely proportional to the growth rate [19]. By altering glucose import capacity in the studied strains, cellular parameters such as *q*_*s*_, *q*_*ac*_ and μ *μ* were simultaneously modified. All of these parameters influence pHN cellular content. The *q*_*s*_ and *μ* displayed by strain WGMCp would represent optimal or near-optimal values for pHN production under the studied conditions which supports the idea that there should be an optimal flux distribution for pDNA synthesis, and that it may be reached not necessarily at very low growth rates. The physiologic state in this strain would be favorable for pHN production by having a relatively low *μ* that increases plasmid content and sufficient glucose import capacity to avoid carbon and energy limitation. When compared to WGMCp, strains WGMp, WGMEp and WHICp, have lower *q*_*s*_. Therefore, it is likely that these strains are energy and carbon-limited, and this state limits plasmid production capacity. A low energy state, resulting from a very low *q*_*s*_ might also explain why strain WHI, when transformed with pHN, did not display growth in minimal medium. This is likely the result of incapacity to cope with the additional demand for precursors and energy required for plasmid replication.

Previous publications have demonstrated an increased capacity for plasmid or recombinant protein production in strains having reduced glucose import capacity. However, in these reports only a few mutant strains were characterized. Therefore, only a small fraction of the potential phenotypic space was sampled. In contrast, in this study, a group of mutants was generated, covering a wide range of glucose import capacity in relatively small steps. By employing this collection of mutants, it was possible to identify a near-optimal glucose import rate for plasmid production capacity under the employed conditions. This *q*_*s*_ value is associated to a physiological state were no overflow metabolism is present and the rate of glucose import still provides sufficient energy and metabolic precursors for cell growth requirements plus the burden of plasmid replication. To our knowledge, there is still no theoretical method to predict such a *q*_*s*_ value, therefore, a combinatorial approach, like the one employed here, should be useful for correlating glucose consumption rate to several physiological and production parameters.

## Conclusions

A group of mutants with modified glucose import capacity was derived from *E. coli* W3110, a robust strain that has been employed as production host for various biotechnological products. This group of mutants affords a large degree of phenotypic diversity that can be explored when searching for specific genotypes leading to improved production capacity. The mutant strains generated and characterized here were employed in experiments for determining the correlation of distinct *q*_*s*_ values to specific physiological and pDNA vaccine production parameters. Although similar data could be obtained by employing chemostat cultures, the use of mutant strains with modified glucose import capacity should simplify subsequent production processes by enabling constant glucose consumption rates in batch cultures.

The characterization of a subset of the generated mutants allowed the identification of a near-optimal *q*_*s*_ value for pHN production. A similar approach can be followed, employing this mutant collection, to identify phenotype(s) leading to the best production parameters for other products such as recombinant proteins.

## Competing interests

The authors declare that they have no competing interests.

## Authors’ contributions

LF, AM and GG participated in the design of this study. LF and LM constructed the strains. LF realized the batch cultures and analyzed the experimental data. LF, AL, OR, AM, FB and GG participated in the analysis of the results as well as in writing and critical review of the manuscript. All authors have read and approved the manuscript.

## Supplementary Material

Additional file 1: Figure S1 Growth profile of W3110 and mutant derivatives in shake flask cultures. Glucose concentration (red squares), biomass concentration (blue circles), acetate concentration (green triangles). A. W3110, B. WG, C. WGX, D. WGB, E. WGE, F. WGM, G. WGMX, H. WGMB, I. WGME, J. WGP, K. WGC, L. WGMP, M. WGMC, N. WHI, O. WHIP, P. WHIC. Click here for file

Additional file 2: Figure S2 Growth profile of W3110 and mutant derivatives transformed with plasmid pHN in shake flask cultures. Glucose concentration (red squares), biomass concentration (blue circles), acetate concentration (green triangles). A. W3110p, B. WGp, C. WGMCp, D. WGMEp, E. WGMp, F. WHICp. Click here for file
