# Supplementary material for: Modification of glucose import capacity in Escherichia coli: physiologic consequences and utility for improving DNA vaccine production
Source: Microb Cell Fact. 2013 May 2;12:42. doi: 10.1186/1475-2859-12-42 (PMC3655049; doi:10.1186/1475-2859-12-42)
Supplement: Additional file 2: Figure S2 — Growth profile of W3110 and mutant derivatives transformed with plasmid pHN in shake flask cultures. Glucose concentration (red squares), biomass concentration (blue circles), acetate concentration (green triangles). A. W3110p, B. WGp, C. WGMCp, D. WGMEp, E. WGMp, F. WHICp. [file 1475-2859-12-42-S2.pptx]

## Slide 1
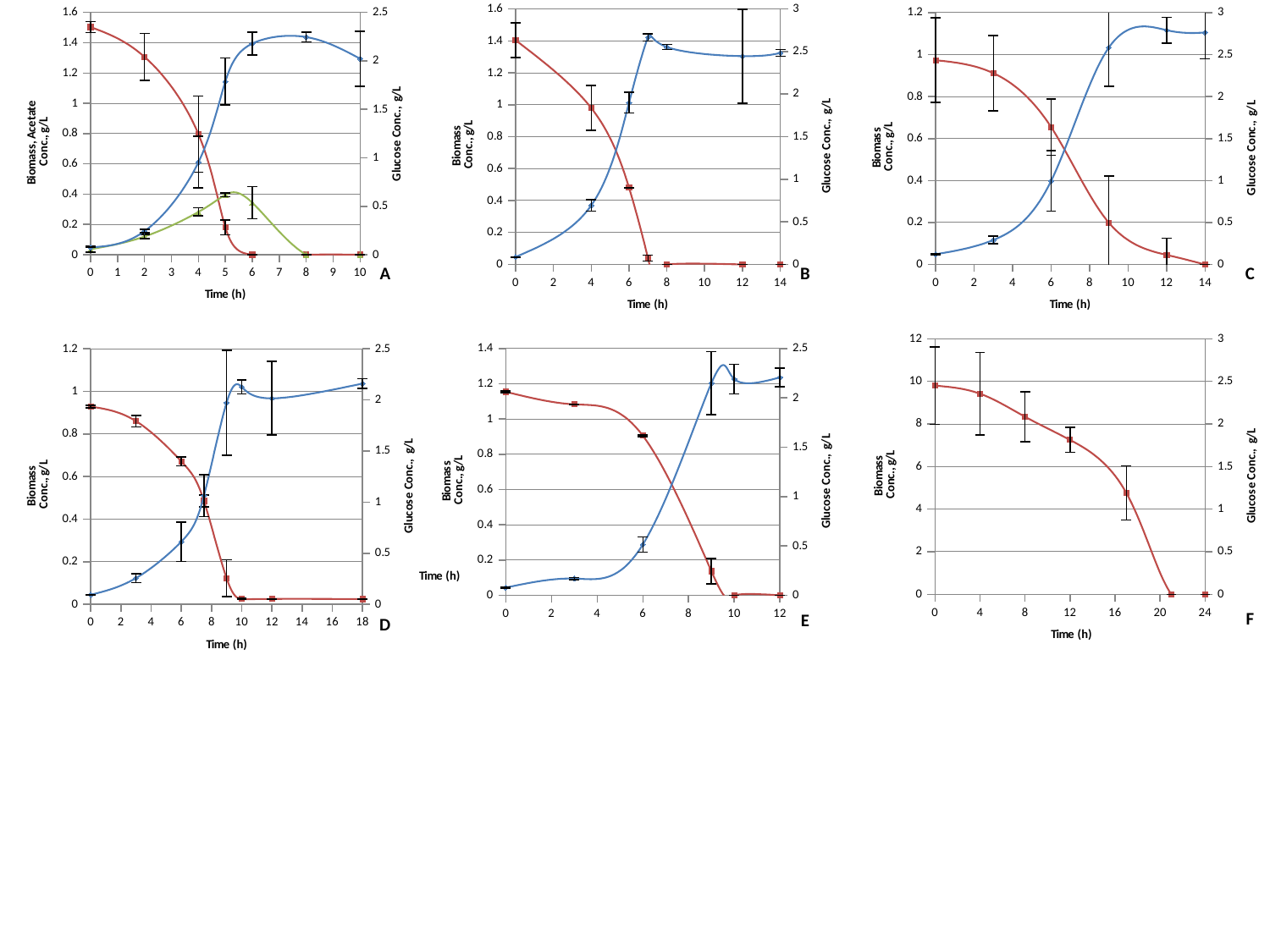

### Chart
| Category | Biomasa | Acetato | Glucosa |
|---|---|---|---|
### Chart
| Category | Biomasa | Glucosa |
|---|---|---|| | | |
| --- | --- | --- |
| | | |
### Chart
| Category | Biomasa | Glucosa |
|---|---|---|A
B
C
### Chart
| Category | promedio | Glucosa |
|---|---|---|
### Chart
| Category | Biomasa | Glucosa |
|---|---|---|
### Chart
| Category | Biomasa | Glucosa |
|---|---|---|F
E
D
